# An Approach for Time Synchronization of Wireless Accelerometer Sensors Using Frequency-Squeezing-Based Operational Modal Analysis

**DOI:** 10.3390/s22134784

**Published:** 2022-06-24

**Authors:** Yi Chen, Xiaoqing Zheng, Yaozhi Luo, Yanbin Shen, Yu Xue, Wenwei Fu

**Affiliations:** 1 College of Civil and Architecture Engineering, Zhejiang University, Hangzhou 310058, China; chenyi123@zju.edu.cn (Y.C.); luoyz@zju.edu.cn (Y.L.); 11812048@zju.edu.cn (Y.X.); fwwll@zju.edu.cn (W.F.); 2 Center for Balance Architecture, Zhejiang University, Hangzhou 310058, China; zheng_xiaoqing@126.com

**Keywords:** time synchronization, wireless sensor, operational modal analysis, frequency-squeezing, structural health monitoring

## Abstract

Wireless sensor networks usually suffer from the issue of time synchronization discrepancy due to environmental effects or clock management collapse. This will result in time delays between the dynamic responses collected by wireless sensors. If non-synchronized dynamic response data are directly used for structural modal identification, it leads to the misestimation of modal parameters. To overcome the non-synchronization issue, this study proposes a time synchronization approach to detect and correct asynchronous dynamic responses based on frequency domain decomposition (FDD) with frequency-squeezing processing (FSP). By imposing the expected relationship between modal phase angles extracted from the first-order singular value spectrum, the time lags between different sensors can be estimated, and synchronization can be achieved. The effectiveness of the proposed approach is fully demonstrated by numerical and experimental studies, as well as field measurement of a large-span spatial structure. The results verify that the proposed approach is effective for the time synchronization of wireless accelerometer sensors.

## 1. Introduction

Structural health monitoring (SHM) systems have been widely implemented in a variety of infrastructures to provide continuous and detailed information to decisionmakers [1,2]. The functionalities of SHM systems are mainly composed of acquiring structural responses, extracting structural features, and assessing structural conditions [3,4,5,6]. In the process, SHM-derived knowledge on structural condition assessment will be affected if the structural dynamic properties are extracted from non-synchronized measurements. For example, a 30 μs synchronization error results in a noticeable error in the modal analysis [7]. This non-synchronization-induced misjudgment will impact the subsequent analysis of an SHM procedure. Therefore, the synchronization of dynamic measured data from different sensors should be guaranteed.

Synchronization discrepancy hardly occurs or it can be easily eliminated by multiple linked data acquisition units (DAU) in a wired sensor network. However, the traditional wired SHM system may become impractical for large-scale civil structures due to strict power supply conditions and large investments in labor and material resources [8,9,10,11]. With the rapid development of wireless communication techniques, wireless sensor networks (WSNs) have been developed to alleviate these limitations. In WSNs, the data transfer speeds of wireless nodes may be different because of the limited bandwidth and low-power radio transceivers [12]. Furthermore, although modern WSNs use clock- management techniques, non-simultaneity in sensor start-up can also result in the time non-synchronization issue. In particular, for passive and low duty cycle wireless sensor nodes, internal clock drift can be caused by temperature change, and which result in hardware start random delay, which poses a significant challenge to the achievement of time synchronization among the wireless sensor nodes. In general, time synchronization is one of the core issues throughout the WSNs community.

Extensive studies have been made to investigate the research topic of time synchronization [13,14,15,16,17,18,19]. The main idea of time synchronization is to determine and align the offset of data. The existing methods can be broadly classified into two categories: the clock synchronization method and the data synchronization method [12,13]. The clock synchronization method exchanges the clock information of sensors and synchronizes them with a global reference time, such as the reference broadcast protocol (RBS) [20], the time-syn protocol for sensor networks (TPSN) [21], and the flood time synchronization protocol (FTSP) [22]. Nevertheless, the non-synchronization issue may still remain in WSNs even when these protocols are employed. Some dynamic measurement errors such as offset, drift, and jitter are commonly found because synchronous sampling is not guaranteed through the use of the clock synchronization method [23]. In contrast, a feasible way to solve this is to eliminate the non-synchronization issue by post-processing the measured data based on the data synchronization method [24]. The core of the data synchronization method is to detect the phase information among the non-synchronicity of measurement data, which can be achieved by the time and frequency domain approaches. For the time domain approaches, Nagayama et al. [25] concluded that only the mode shapes rather than natural frequencies and damping ratios will be affected by non-synchronization dynamic measurements, which means that the phase difference caused by time delay strongly influenced the mode shapes. To correct time-delay-induced errors, Lei et al. [15] estimated the time delay by fitting the measured data to an autoregressive model (ARX) or an average autoregressive model. Zhou et al. [26] corrected the time delay by a state-space (SS) equation model combined with a data-driven stochastic subspace identification (data-driven SSI) method to calculate the mean phase deviation. These algorithms need to determine a reasonable number of model orders to obtain the real-time lag information and are too computationally complex to achieve rapid evaluation. Zhang et al. [27] proposed an output correlation-based approach, which mainly focused on analyzing the influence on the mode shape estimations with small delays. Their utility may be diminished if these methods are used to realign non-synchronous measurement data collected at different locations within a structure. The frequency-domain methods are based on the correlation between the Fourier amplitudes of each response and the time lags. Dragos et al. [28,29] estimated the time delays with a high sampling rate by using the phase information of the Fourier spectrum of acceleration data from different sensor nodes. Zhou et al. [30] calculated the slope of the phase angle curve by estimating the cross power spectral density (CPSD) to determine the lag. Bernal [31] introduced an approach to minimize the errors caused by asynchronicity based on shifting the signals in the time domain. The phases of the fundamental eigenvector estimated from the spectral density are zero, which shows that signal realignment is preferred to the correction of the eigenvectors.

Despite the developments of time-synchronization methods, the existing method seldom considered measuring noise. In practice, the field-measured data of a real structure is inevitably subjected to ambient noise. In particular, for large-span structures with large stiffness, the signal-to-noise ratio (SNR) will be rather small. In this case, the estimation of the modal phase will be greatly affected, which may cause failure when using the existing methods. To cope with it, this study develops a frequency squeezing-based frequency-domain decomposition method (FSP-FDD) for time synchronization. The FSP-FDD exploits the characteristics of multi-channel dynamic responses on the spectral concentration distribution. It can reduce the uncertainty of peak selection caused by noise by appropriately squeezing the frequency, thereby getting a more accurate estimate of the modal phase. Then, the lags are determined based on the relationship between the lags and the modal phase. The effectiveness of the presented approach is firstly demonstrated by numerical simulation and experimental study. Subsequently, the practicality of the approach is further validated using the field-measured non-synchronous dynamic data of a cable-net structure subjected to strong wind.

## 2. Time Synchronization Approach

**Figure 1:**
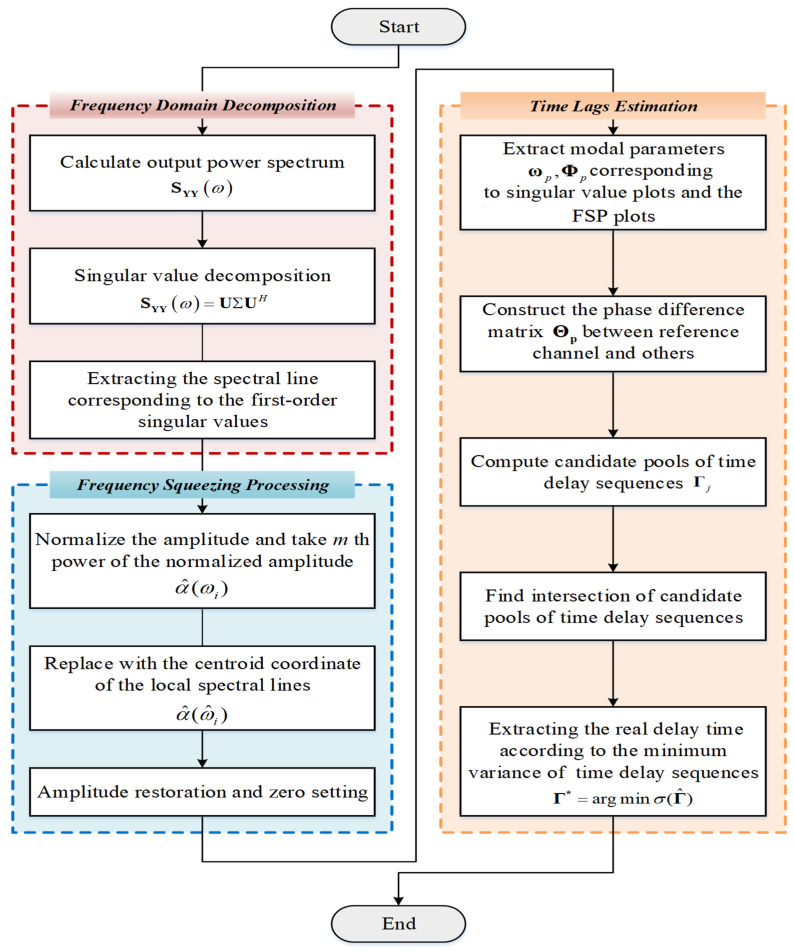
Flowchart of non-synchronous measurement correction with FSP-FDD.

Generally, the dynamic behavior of civil structures is described as a linear system with a light and proportional damping assumption. Under this assumption, the mode shapes of structures can be accurately extracted from synchronized measurements. The different degrees of freedom (DOFs) reach the furthest and the equilibrium position simultaneously. Accordingly, the mode shape components of one certain mode extracted between any two synchronous signals lie on the real axis in a complexity plot, i.e., the phase angles are equal to 0° (in-phase) or 180° (out of phase) [29]. When these two dynamic response measurements are non-synchronous, there is a mapping relationship between the relative lag and their phase angles. Then, the delays between the signals can be obtained based on the deviation between the actual and ideal phase angles. The framework of the presented synchronization algorithm is shown in Figure 1.

Considering two non-synchronous responses $y_{1}(t)$ and $y_{2}(t-\tau_{12})$ collected by WSNs, where $\tau_{12}$ denotes the relative time lag between two responses, their Fourier transforms are

(1) $$Y_{1}(\omega )=\int_{-\infty }^{\infty }e^{-i\omega t}y_{1}(t)dt$$

(2) $$Y_{2}^{\prime }(\omega )=e^{i\omega \tau_{12}}\int_{-\infty }^{\infty }e^{-i\omega t}y_{2}(t-\tau_{12})dt=e^{i\omega \tau_{12}}Y_{2}(\omega )$$

where ω and *i* are the circular frequency and the imaginary unit, respectively. The cross power spectral density (CPSD) is a fundamental tool for modal identification, which measures the distribution of power for the pair of signals across a frequency spectrum. Through CPSD, the relationship between these two time-domain signals can also be expressed as

(3) $$S_{12}^{\prime }(\omega )=Y_{1}^{*}(\omega )Y_{2}^{\prime }(\omega )=e^{i\omega \tau_{12}}Y_{1}^{*}(\omega )Y_{2}(\omega )$$

where the superscript * refers to the complex conjugate operator. Comparison of Equations (2) and (3) show that the time-delayed response results in a rescaling within the frequency domain by multiplying with $e^{i\omega \tau_{12}}$. Correspondingly, it also describes that the time lags will lead to a shifted phase $\theta_{12}$ in a polar form where $\theta_{12}=\omega \tau_{12}$. Therefore, the relationship between the two outputs can be extended to the frequency spectra for estimation of the relative time lags.

For better illustration, dynamic monitoring data of one channel are selected as a reference, then the delays between the referenced channel and the rest channels can be uniquely quantified. The vectors of the rest channels can be written as

(4) $$Y{\left(t\right)}_{g\times N}={\left[y_{1}(t),y_{2}(t-\tau_{12}),\cdots ,y_{g}(t-\tau_{1g})\right]}^{T}$$

where $\tau_{12},\cdots ,\tau_{1g}$ are the time lags between the referenced and rest channel, and *N* is the length of the signal, *g* is the number of the output channel; Similar to Equation (3), the CPSD matrix is introduced as follows.

(5) $$S_{YY}(i\omega )=\left[\begin{array}{ccc} S_{11}(i\omega ) & \cdots & e^{-i\omega \tau_{1g}}S_{k1}(i\omega )\\ \vdots & \ddots & \vdots \\ e^{i\omega \tau_{1g}}S_{g1}(i\omega ) & \cdots & S_{gg}(i\omega_{p}) \end{array}\right]$$

Assuming that one mode is dominant at the frequency $\omega_{p}^{\left(k\right)}$ associated with the resonance of *k*-th mode. Then, by taking the singular value decomposition (SVD) of the CPSD matrix, the CPSD matrix can be approximated to a 1-rank matrix, rewritten as

(6) $$S_{YY}(i\omega_{p}^{\left(k\right)})=\left[\begin{array}{ccc} S_{11}(i\omega_{p}^{\left(k\right)}) & \cdots & e^{-i\omega_{kp}\tau_{1g}}S_{k1}(i\omega_{p}^{\left(k\right)})\\ \vdots & \ddots & \vdots \\ e^{i\omega_{kp}\tau_{1g}}S_{g1}(i\omega_{p}^{\left(k\right)}) & \cdots & S_{gg}(i\omega_{p}^{\left(k\right)}) \end{array}\right]\approx \sigma_{1}u_{1}u_{1}{}^{H}\;\;\;\omega \to \omega_{p}^{\left(k\right)}$$

where the superscript *H* refers to the complex conjugate transpose operator; and $u_{1}$ is the first singular vector representing the estimation of the *k*-th mode shape

(7) $$\Phi^{\left(k\right)}=u_{1}(\omega_{p}^{\left(k\right)})=\left[1,\cdots ,e^{i\omega_{kp}\tau_{1g}}\right]{\bar{\Phi }}^{\left(k\right)}$$

where ${\bar{\Phi }}^{\left(k\right)}$ is the *k*-th mode shape extracted from the ideal synchronization signals. Considering the assumption of proportional damping, the mode shape vectors are real-valued. Without the loss of generality, suppose that $\phi_{1n}^{\left(k\right)}$ and ${\bar{\phi }}_{1n}^{\left(k\right)}$ are *n*-th components of $\Phi^{\left(k\right)}$ and ${\bar{\Phi }}^{\left(k\right)}$, respectively. Their one-to-one relationship between $\phi_{1n}^{\left(k\right)}$ and ${\bar{\phi }}_{1n}^{\left(k\right)}$ is conducted, given by

(8) $${\bar{\phi }}_{1n}^{\left(k\right)}=\mathrm{sgn}\left(\cos\theta_{1n}^{\left(k\right)}\right)\left|\phi_{1n}^{\left(k\right)}\right|$$

Accordingly, the time lag $\tau_{1n}$ can be written as follows

(9) $$\tau_{1n}=\frac{\theta_{1n}^{\left(k\right)}}{\omega_{p}^{\left(k\right)}}$$

where sgn means the sign function; and $\theta_{1n}^{\left(k\right)}$ denotes the shifted phase in one period ($T_{p}^{\left(k\right)}=2\pi /\omega_{p}^{\left(k\right)}$) between the referenced channel and the rest channel *n* under the *k*-th mode, that is $\theta_{1n}^{\left(k\right)}=\theta_{1}^{\left(k\right)}-\theta_{n}^{\left(k\right)}$. Additionally, the phase angle for the *k*-th mode shape vector can be obtained

(10) θj(k)={arctan(Re(ϕj(k))Im(ϕj(k)))if arctan(Re(ϕj(k))Im(ϕj(k)))≥0arctan(Re(ϕj(k))Im(ϕj(k)))+πif arctan(Re(ϕj(k))Im(ϕj(k)))<0j∈[1,⋯,m,⋯,g]

Considering that the lag $\tau_{1n}$ may exceed the period, Equation (9) is rewritten in a generalized form

(11) $$\tau_{1n}=\frac{\theta_{1n}^{\left(k\right)}}{\omega_{p}^{\left(k\right)}}+k_{1n}^{\left(k\right)}\pi \;\;\;k_{1n}^{\left(k\right)}\in Z$$

Similarly, a set of *g* − 1 equations with (2*g* − 2) unknown parameters $k_{12}^{\left(k\right)},\cdots ,k_{1m}^{\left(k\right)},\cdots ,k_{12}^{\left(k\right)}$ are obtained:

(12) $$\{\begin{array}{c} \tau_{12}=\frac{\theta_{12}^{\left(k\right)}}{\omega_{p}^{\left(k\right)}}+k_{12}^{\left(k\right)}\pi \\ \vdots \\ \tau_{1n}=\frac{\theta_{1n}^{\left(k\right)}}{\omega_{p}^{\left(k\right)}}+k_{1n}^{\left(k\right)}\pi \\ \vdots \\ \tau_{1g}=\frac{\theta_{1g}^{\left(k\right)}}{\omega_{p}^{\left(k\right)}}+k_{1g}^{\left(k\right)}\pi \end{array}\;k_{12}^{\left(k\right)},\cdots ,k_{1n}^{\left(k\right)},\cdots ,k_{1g}^{\left(k\right)}\;\in Z$$

Obviously, Equation (12) is definitely underdetermined because the number of unknowns (2*g* − 2) exceeds the number of equations (*g* − 1). Hence, additional information needs to be introduced. Suppose that *M* (*M* > 1) modes have been identified by FDD technique [32]. A shifted-phase matrix is built as

(13) $$\Theta =\left[\begin{array}{ccc} \theta_{12}^{\left(1\right)} & \cdots & \theta_{12}^{\left(M\right)}\\ \vdots & \ddots & \vdots \\ \theta_{1g}^{\left(1\right)} & \cdots & \theta_{1g}^{\left(M\right)} \end{array}\right]$$

Accordingly, rewriting Equation (12) in matrix form yields

(14) $$\Gamma =\left[\begin{array}{ccc} \frac{\theta_{12}^{\left(1\right)}+k_{12}^{\left(1\right)}\pi }{\omega_{p}^{\left(1\right)}} & \cdots & \frac{\theta_{12}^{\left(M\right)}+k_{12}^{\left(M\right)}\pi }{\omega_{p}^{\left(M\right)}}\\ \vdots & \ddots & \vdots \\ \frac{\theta_{1g}^{\left(1\right)}+k_{1g}^{\left(1\right)}\pi }{\omega_{p}^{\left(1\right)}} & \cdots & \frac{\theta_{1g}^{\left(M\right)}+k_{1g}^{\left(M\right)}\pi }{\omega_{p}^{\left(M\right)}} \end{array}\right]k_{1j}^{\left(i\right)}\in Z$$

where the superscript in Equation (14) denotes the number of the identified mode; and $\omega_{p}^{\left(1\right)},\cdots ,\omega_{p}^{\left(M\right)}$ are the selected frequencies associated with the peak of resonance of the identified modes. Since the values $k_{1j}^{\left(i\right)}$ can only be taken in the integer domain, this greatly narrows the scope of the solution. For each row of the matrix in Equation (14), through a series trial of $k_{1j}^{\left(i\right)}$ where j∈[1,⋯,g] and i∈[1,⋯,M], then, a candidate pool for the actual time lags can be easily determined as

(15) $$\hat{\Gamma }=\left[{\hat{\tau }}_{12},\cdots ,{\hat{\tau }}_{1g}\right]$$

Obviously, there exists more than one possible candidate for each relative time lag. Therefore, it necessitates a solution to determine the optimal time lags. The final time lag can be estimated based on the lowest standard deviation of Equation (15). In other words, the lags are often around the expectation of the set of final lags, which yields the lowest standard deviation. The actual lags $\tau_{12},\cdots ,\tau_{1g}$ can be estimated as follows

(16) $$\Gamma^{*}=\underset{k_{1j}^{i}\in Z}{\arg\min}\sigma (\hat{\Gamma })$$

(17) $$\left[\tau_{12},\cdots ,\tau_{1g}]=E(\Gamma^{*}\right)$$

It is noteworthy that the accuracy of the identified natural frequencies and non-synchronous mode shapes play a primary role in lag estimation. However, in the process of estimating the CPSD by FDD, the sampled signal duration is limited and accompanied by various noises. When the SNR is low, there are multiple potential candidate peaks caused by the noise, which increases the uncertainty in the peak selection. As a result, the presence of noise affects the estimation of the modal parameter, especially in weak excitation. Thus, it is necessary to minimize the influence of the noise.

**Figure 2:**
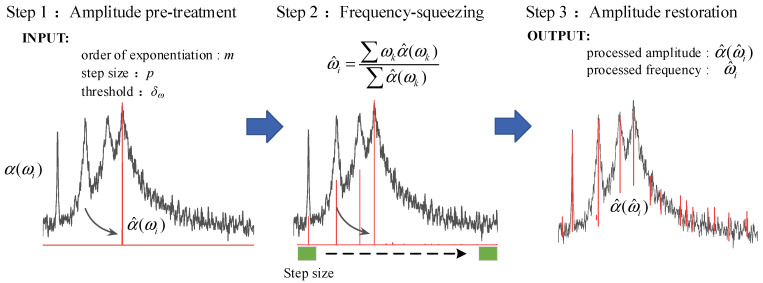
A schematic diagram of FSP.

Learning from the stabilization diagram [32], the actual modes can be identified from alignments of stable poles since the spurious modes tend to be more scattered when increasing model orders. Given this, this study introduces frequency-squeezing to improve the readability of the power spectrum/singular value (SV) spectrum representation. The FSP is based on shifting the local spectrum shape to its nearby natural frequency without changing its magnitude [33]. A schematic diagram of FSP is depicted in Figure 2, which consists of three main steps detailed as follows.

Step 1: Amplitude pretreatment of the first-order singular spectrum. First, the spectrum amplitude is normalized to [0, 1]. Then, the amplitude is “shaped” by taking the *m*-th power of the normalized amplitude for subsequent processing. The amplitude pretreatment can be written as

(18) $$\bar{\alpha }(\omega_{i})=\alpha (\omega_{i})/\max(\alpha (\omega_{i}))\;\;\;i=1,2,\ldots ,K$$

(19) $$\hat{\alpha }(\omega_{i})={\left(\bar{\alpha }(\omega_{i})\right)}^{m}\;$$

where $\alpha (\omega_{i})$ and $\hat{\alpha }(\omega_{i})$ are the spectral amplitudes before and after processing, respectively; $\omega_{i}$ is the sampling frequency point from a vector $\omega =\left[\omega_{1},\omega_{2},\cdots ,\omega_{K}\right]$; *m* can be set as integer multiples of 10 to reduce rapidly the amplitude of the peak nearby.

Step 2: Frequency-squeezing for the pretreated signal. Considering the continuous 2*p* + 1 (*p* ≥ 1) spectral lines $\hat{\alpha }(\omega_{i-p}),\cdots ,\hat{\alpha }(\omega_{i}),\cdots ,\hat{\alpha }(\omega_{i+p})$, the frequency ${\hat{\omega }}_{i}$ is replaced by the centroid coordinate of a graph, which is composed of these spectral lines and the frequency $\omega_{i-p},\cdots ,\omega_{i},\cdots ,\omega_{i+p}$, given by

(20) $${\hat{\omega }}_{i}=\{\begin{array}{l} \frac{\sum_{k=1}^{i+p}\omega_{k}\hat{\alpha }(\omega_{k})}{\sum_{k=1}^{i+p}\hat{\alpha }(\omega_{k})}\;\;\;i=1,\cdots ,p\\ \frac{\sum_{k=i-p}^{i+p}\omega_{k}\hat{\alpha }(\omega_{k})}{\sum_{k=i-p}^{i+p}\hat{\alpha }(\omega_{k})}\;\;\;i=p+1,\cdots ,K-p\\ \frac{\sum_{k=i-p}^{K}\omega_{k}\hat{\alpha }(\omega_{k})}{\sum_{k=i-p}^{K}\hat{\alpha }(\omega_{k})}\;\;\;i=K-p+1,\cdots ,K \end{array}$$

where *p* and *K* are the user-specified step and the signal length, respectively. Then, repeat the step until the convergence criterion is satisfied. The convergence criterion is defined as.

(21) $${\left\|{\hat{\omega }}^{s+1}-{\hat{\omega }}^{s}\right\|}_{2}/K<\delta$$

where *s* is the number of iteration.

Step 3: Amplitude restoration and zero settings. Since the magnitude of the amplitude is normalized in Step 1, the accurate amplitude information should be retained. The original amplitude vector is assigned to the newly generated frequency vector in the order of subscripts, and the amplitude between the edges and the cluster of aggregated frequency points is set to zero, which can be written as

(22) $$\hat{\alpha }({\hat{\omega }}_{i})=\{\begin{array}{ll} 0 & i\in \Omega =\left\{1,K\right\}\cup \left\{{\hat{\omega }}_{i+1}-{\hat{\omega }}_{i}>\delta_{\omega }\right\}\\ \alpha (\omega_{i}) & i\in \left\{1,2,\cdots ,K\right\}\backslash \Omega \end{array}$$

where Ω is the set of frequency subscripts corresponding to the set of zero amplitude. $\delta_{\omega }<\Delta \omega =\omega_{i+1}-\omega_{i}$ is the indicator to determine the abnormal frequency, which is suggested to be set as 0.01 or 0.001 times Δω.

In conclusion, the FSP technique artificially changes the orthogonality characteristics of the basis vector after the Fourier transform of the signal. It highlights the natural frequency, which can serve as the referenced frequency for peak selection in the FDD method. 

## 3. Evaluation of FSP-FDD Method with Non-Synchronization Responses

### 3.1. Numerical Simulation

#### 3.1.1. Structural Description

**Figure 3:**
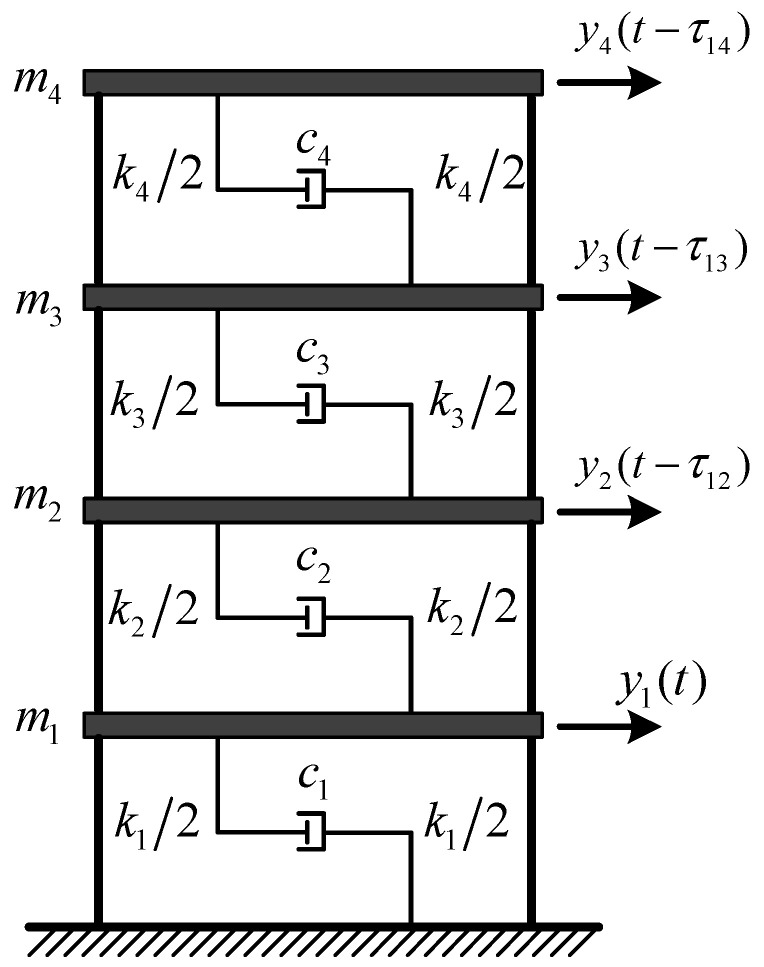
A linear time-invariant model of a four-story building.

A linear time-invariant model of a four-story building (Figure 3) is used as tested. Each floor is represented as masses $m_{i}$ (i∈(1,⋯,4)) interconnected with springs $k_{i}$ and dampers $c_{i}$. The weight of each mass, the constants of lateral shear stiffness $k_{i}$, and the damping coefficients $c_{i}$ between adjacent floors are 10 kg, 1000 kg/m, and 10 N·s/m, respectively. The mass matrix **M**, stiffness matrix **K**, and damping matrix **C** can be expressed as

(23) $$M=\left[\begin{array}{cccc} 10 & 0 & 0 & 0\\ 0 & 10 & 0 & 0\\ 0 & 0 & 10 & 0\\ 0 & 0 & 0 & 10 \end{array}\right]\;,\;K=\left[\begin{array}{cccc} 2 & -1 & 0 & 0\\ -1 & 2 & -1 & 0\\ 0 & -1 & 2 & -1\\ 0 & 0 & -1 & 2 \end{array}\right]\times 10^{3}\;,\;C=\left[\begin{array}{cccc} 20 & -10 & 0 & 0\\ -10 & 20 & -10 & 0\\ 0 & -10 & 20 & -10\\ 0 & 0 & -10 & 20 \end{array}\right]$$

Each floor is excited by a stationary, zero-mean, Gaussian white noise. By adding state noise N(0,0.01) and output noise N(0,0.001) into the structure, then the structural responses under white noise excitation are simulated (SNR = 20 dB). All the responses have a duration of 100 s and the sampling rate is 50 Hz. Suppose that each floor has an independent acquisition unit for response collection. Four sets of dynamic responses with different lags are set intentionally to assess the impact of the non-synchronization on modal identification. The first channel is set as the referenced channel, and the relative time delays of other channels are shown in Table 1, where the positive sign indicates that the time is behind the reference timeline and vice versa.

#### 3.1.2. Method Validation

**Figure 4:**
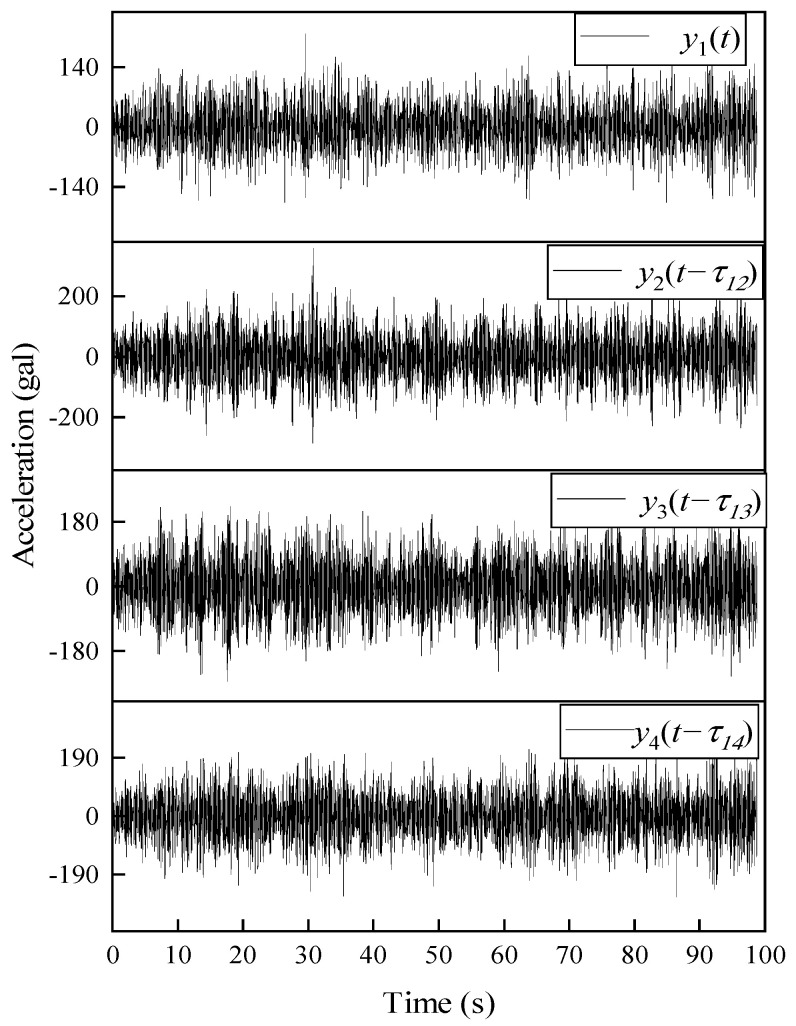
Artificially misaligned accelerations response of the LTI system.

**Figure 5:**
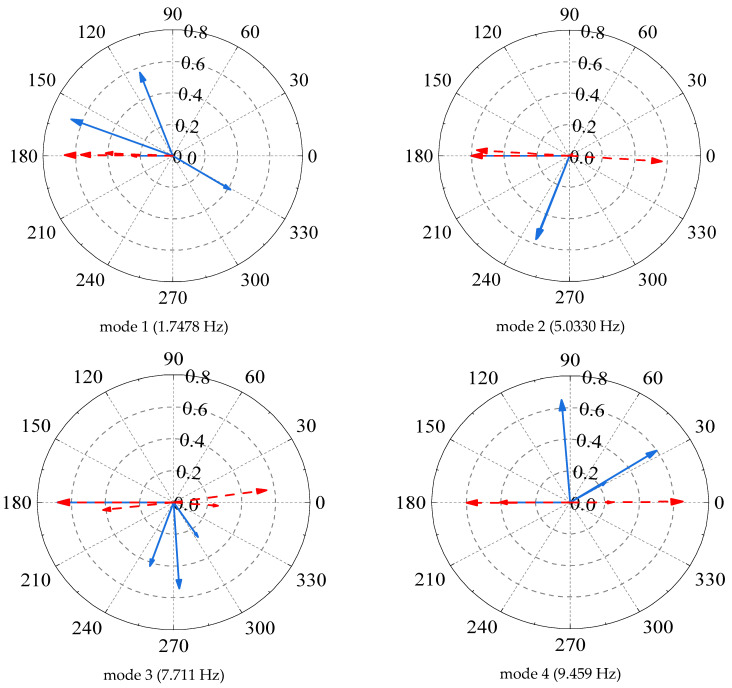
Comparison of theoretical and estimated mode shapes in complexity. (The red dash lines refers to the theoretical mode shapes whereas the blue lines refers to the estimated mode shapes).

Figure 4 shows the unprocessed acceleration response measurements. For showing the impact of time delay on the mode shapes, theoretical mode shapes computed with synchronous data are introduced. The modal phase angles obtained from the theoretical mode shapes and the ones identified by FDD using non-synchronous data are plotted in the polar form shown in Figure 5. It is noteworthy that the phase of the theoretical mode shapes (red dash lines) lies on nearly straight lines, as expected. The phases of the 1st mode are moving in phase whereas the rest of modes are moving out of phase. However, the mode shapes identified from non-synchronous data are highly complex, which could lead to wrong conclusions such as high levels of nonlinearities or large damping. Meanwhile, by a complex-to-real conversion of mode shapes, it is found that the amplitude of identified mode shapes is smaller than the theoretical results. One primary reason for it is that the amplitudes are rescaled by a factor caused by phase shift. Therefore, the time delay in dynamic measurement greatly affects the identification of the modal parameters. 

**Figure 6:**
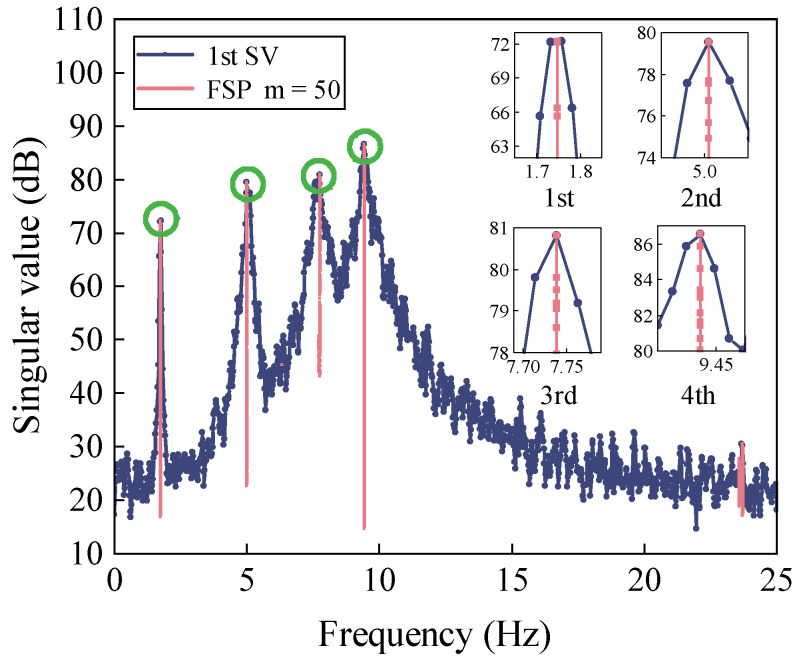
First-order singular value plots obtained from FSP-FDD.

In order to find actual lags, Figure 6 displays the first-order singular value obtained from FDD-FSP. Recalling Equation (20), the computational parameters are as follows: the step size (2*p* + 1) is set as 121, the order of exponentiation (*m*) is set as 50, the frequency convergence threshold (*δ*) is set as 1 × 10^−6^, and the total iteration number is 1000. The spectrum is concentrated at the true position of natural frequency. The advantage of FSP is the reduction of distortion in the target frequency pickup and the improved estimation accuracy of the delay (Equation (14)). To exemplify this, a zoomed-in view of Figure 6 is shown. It is clear that the original first-order spectral line moves to the target peak where the 1st to 4th order frequencies are well-reflected.

After performing the FSP-FDD, the candidate pool of lags can be easily conducted. Taking an explanatory example of the relative delay between channel 1 and channel 2, the candidate lags can be written as

(24) $$\{\begin{array}{l} \tau_{12}^{\left(1\right)}=0.0494+0.2844k_{12}^{\left(1\right)}\\ \tau_{12}^{\left(2\right)}=-0.0381+0.0999k_{12}^{\left(2\right)}\\ \tau_{12}^{\left(3\right)}=0.0202+0.0646k_{12}^{\left(3\right)}\\ \tau_{12}^{\left(4\right)}=0.0252+0.0531k_{12}^{\left(4\right)} \end{array}$$

**Figure 7:**
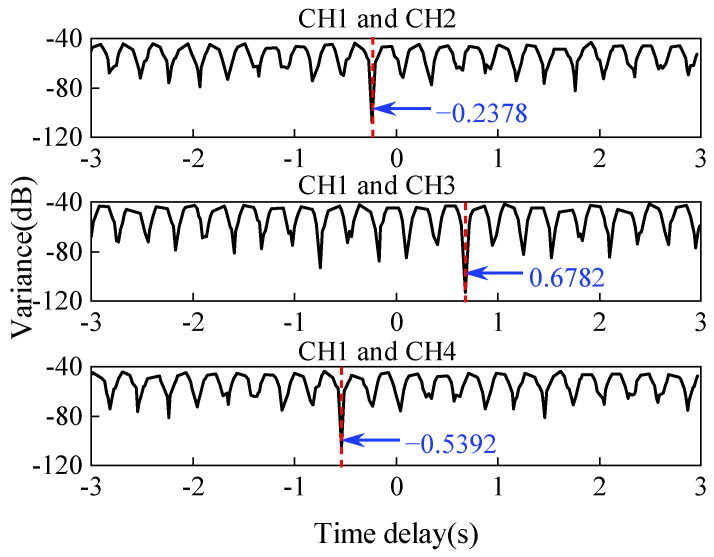
Relationships between delay and variance of the candidate pool time lags.

**Figure 8:**
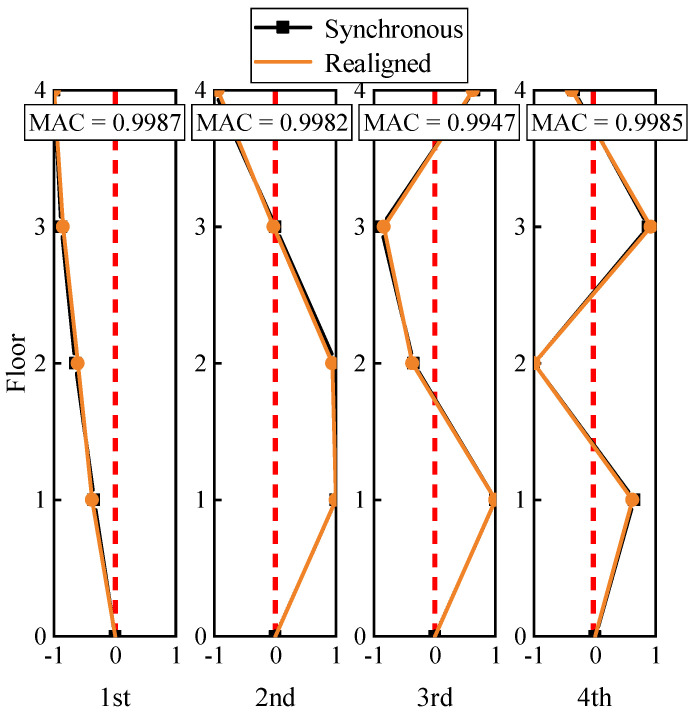
Mode shapes of lateral bending modes in synchronous and realigned.

When assigning −1, −2, −4, −5 to $k_{12}^{\left(1\right)},k_{12}^{\left(2\right)},k_{12}^{\left(3\right)},k_{12}^{\left(4\right)}$ through trial computation, respectively, the relative delay $\tau_{12}^{\left(1\right)},\tau_{12}^{\left(2\right)},\tau_{12}^{\left(3\right)},\tau_{12}^{\left(4\right)}$ is −0.2350, −0.2379, −0.2382, −0.2403, which has the lowest standard deviation of the relative delay set. And the mean of this set (−0.2378) is nearly equal to the preset delay (−0.2400). Considering that the relative time lag should satisfy the integral multiple of sampling interval (0.0200 s), the time lag is obtained as −0.2400 s. Similarly, the detected lag of the rest of channels is also solved and shown in Figure 7. Finally, the modal parameters are re-identified using the realigned dynamic response (Figure 8). The modal assurance criterions (MACs) between the mode shapes obtained from the realigned and synchronous data can be written as

(25) $$\mathrm{MAC}(\Phi_{i}^{t},\Phi_{j}^{m})=\frac{\left|{\left(\Phi_{i}^{t}\right)}^{H}\left(\Phi_{j}^{m}\right)\right|}{{\left(\Phi_{i}^{t}\right)}^{H}\left(\Phi_{i}^{t}\right){\left(\Phi_{j}^{m}\right)}^{H}\left(\Phi_{j}^{m}\right)}$$

where $\Phi_{i}^{t}$ and $\Phi_{j}^{m}$ refers to the mode shape vector extracted from the realigned and the previous synchronous responses, respectively. The MACs of the mode shapes are near 1 (Figure 8), which shows that the mode shapes obtained by the processed data match well with the theoretical ones. 

### 3.2. Experimental Study

#### 3.2.1. Structural Description

**Figure 9:**
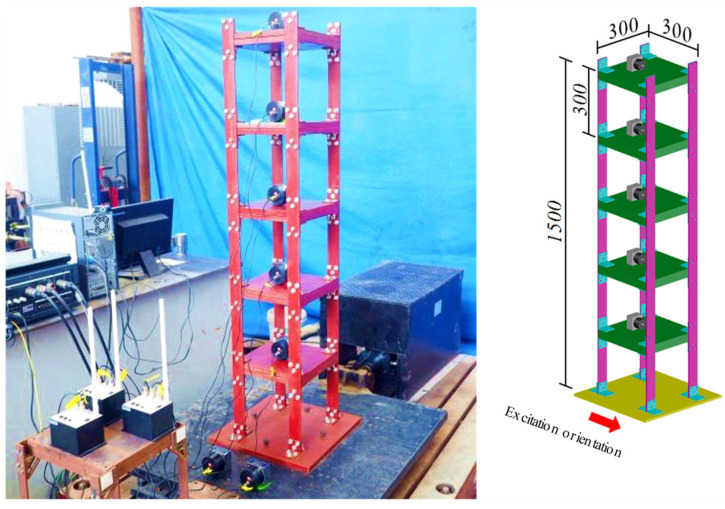
Frame and sensor configuration of the shake table test.

**Figure 10:**
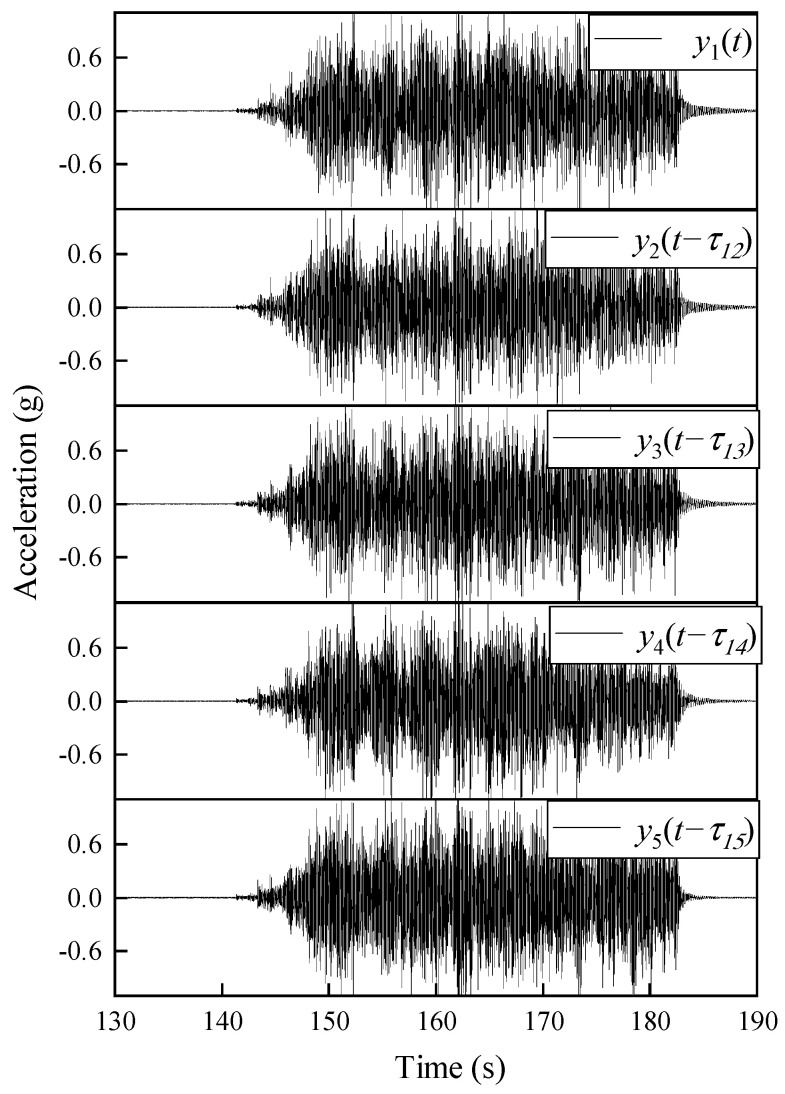
Artificially misaligned accelerations response under shake-table excitation.

As shown in Figure 9, the shake table test model of a five-floor steel frame is utilized to further demonstrate the effectiveness of the proposed approach. The geometrical and material properties of this structure are: the floor height is h = 300 mm, the cross-section of the columns is A = 50 × 5 mm, the elastic modulus E = 206 GPa, the Poisson’s ratio υ = 0.31, and the mass density is 7850 kg/m^3^. Each floor consists of two steel plates with a size of 300 × 300 × 20 mm, connected to the columns by eight angle-iron brackets. The layout of the wireless acceleration sensors (WASs) is also depicted in Figure 9. The channel number of these WASs is the same as the floor numbers. The DAU contains two 3-channels and a 24-bit analog-to-digital conversion (ADC). This frame was excited by the Hollister earthquake [34]. The sampling frequency was set to 128 Hz. The data collected during the warming up of the shake table is discarded, and total 15,360 discrete data during the earthquake excitation were acquired. The main measurement responses are shown in Figure 10.

#### 3.2.2. Method Validation

As outlined in Table 2, three cases of relative time delays were artificially injected into the acceleration data, then the relative percentage error (RPE) between estimated time lags and exact time lags also were calculated for evaluating the accuracy of time delay estimation.

**Figure 11:**
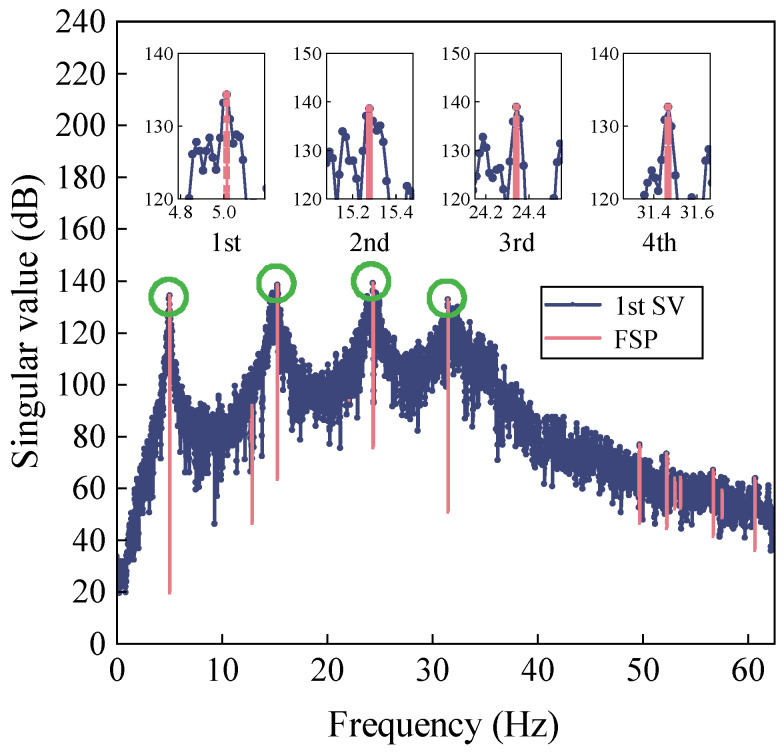
First-order singular value plots obtained from FSP-FDD.

**Figure 12:**
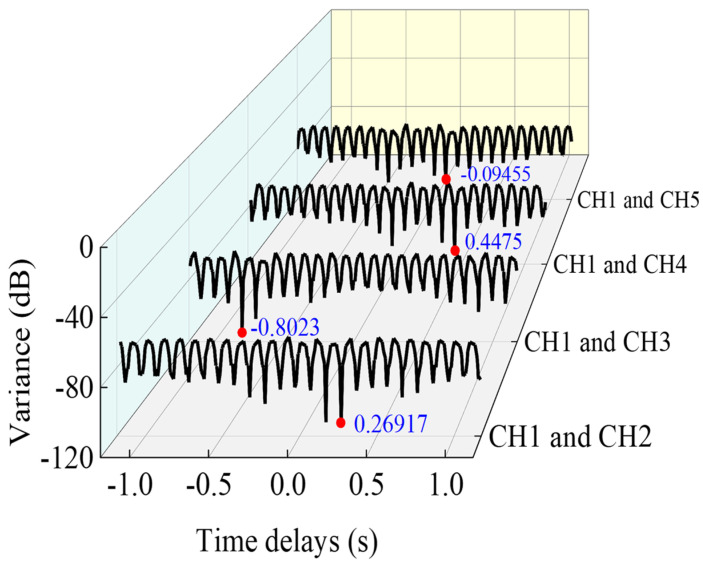
Relationships between delay and variance of candidate pool time lags.

Time delays in each case are estimated by using the proposed time synchronization approach. Although affected by random measurement noise, the first four modes can be easily identified through the reference peak position by FSP (Figure 11). The first-order spectrum is smoothly concentrated at the target frequencies. Then, the relationship between the candidate pool of relative time lags and its standard deviation is obtained by minimizing the standard deviation (Equation (16)), as is depicted in Figure 12. 

**Figure 13:**
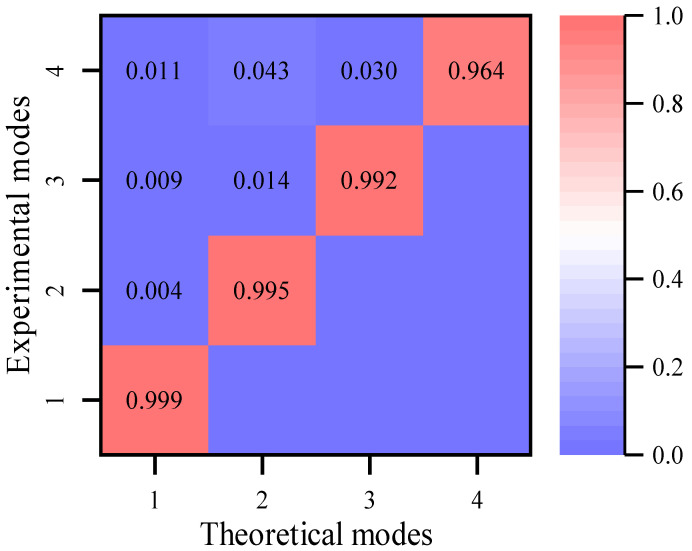
MAC after time lag correction.

As is shown in Figure 13, the value of the MAC matrix indicates that the mode shapes obtained from the realigned responses are very similar to those obtained from the synchronous data. In particular, in the synchronous case (Case 3), the estimated lag is near zero. Hence, the relative lags of the experimental data are precisely estimated by the proposed approach, which validates the effectiveness of the proposed approach.

## 4. Verification with Monitoring Data

### 4.1. Description of NSSO and Its Monitoring System

**Figure 14:**
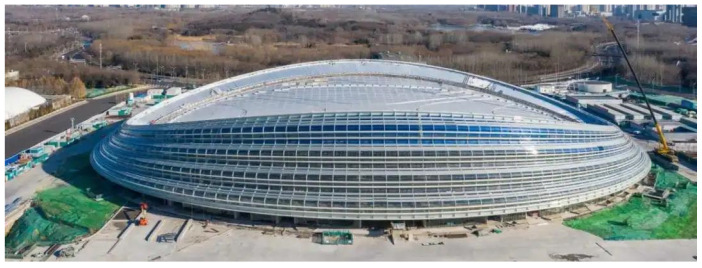
National Speed Skating Oval (NSSO).

**Figure 15:**
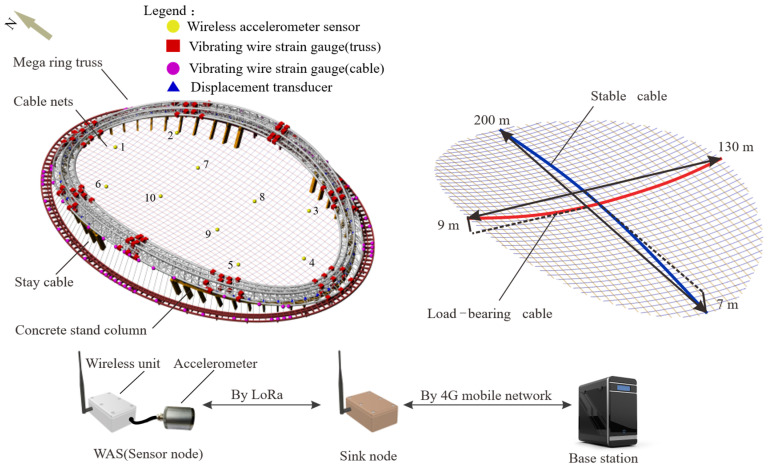
Layout of the sensors and monitoring procedure based on WSN.

To further investigate the performance of the presented approach, the field-measured data of the National Speed Skating Oval (NSSO) is adopted. The NSSO (Figure 14), located in the Beijing Olympic Park, China, was built for hosting the speed skating events during the 2022 Beijing Winter Olympics, with a span of 220 m × 153 m. It comprises four main parts: the saddle-shaped cable net, the mega ring truss, the concrete stand columns, and the stay cables. As shown in Figure 15, the cable net consists of stable cables and load-bearing cables and has a span of 200 m × 130 m.

A customized wireless SHM system designed by Zhejiang University Space Structure Center is implemented on the structure [35,36]. This wireless SHM system consists of more than 300 sensors. Each WAS, composed of a tri-axis accelerometer and a wireless unit, is deployed at the cables to obtain the modes of interest. An idle-wakeup mechanism is used in this wireless SHM system to reduce energy consumption. The measured data from all WAS is transmitted to the sink nodes by Long Range Transmission (LoRa), which is a proprietary low-power wide-area network modulation technique. Although this wireless system promotes high flexibility and less implementation cost, it also brings the time synchronization challenge. 

### 4.2. Sensor Attitude Adjustment for Modal Identification

**Figure 16:**
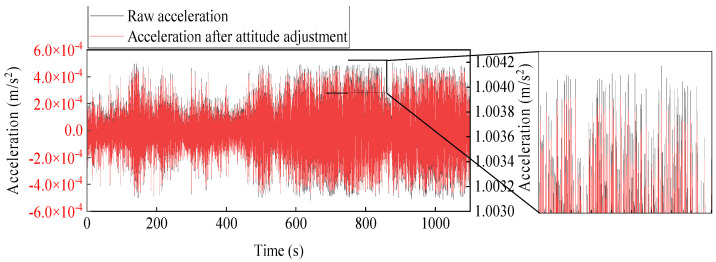
Raw acceleration and adjusted acceleration example from WAS-1.

**Figure 17:**
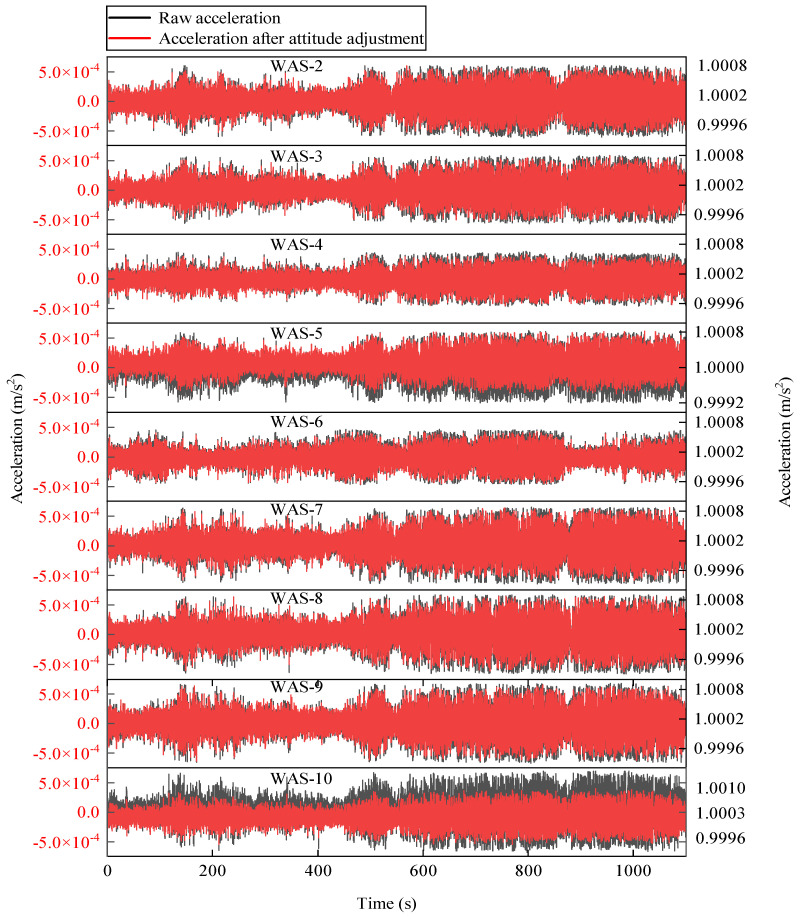
Processed acceleration sets after sensor attitude adjustment.

Acceleration measurements were automatically recorded by the monitoring system during strong wind on 18 May 2021. The acceleration WAS-1, shown in Figure 16, was chosen as the reference. Note that these measured 3-dimensional accelerations contain gravity information. The mean components in the *x*-axis, *y*-axis, and *z*-axis of WAS-1 are −0.094 g, −0.105 g, and 1.003 g, respectively. However, the ideal components should be 0 g, 0 g, 1 g when the sensor coordinate system coincides with the Earth coordinate system. This indicates that the sensor attitude is changed from the instrumentation plan due to the curvature changes of cable or installation deviation. The sensor attitudes can be corrected into the earth coordinate system by applying the coordinate transformation matrix [37] to improve the accuracy of the identified mode shapes. The proposed measurement responses after the sensor attitude correction are depicted in Figure 17. 

### 4.3. Analysis Results

As mentioned above, the time synchronicity cannot be secured with long-distance and multi-hop communication in the WSN system. The proposed approach is used to detect the relative time delays between different response channels. 

The FE model of the NSSO was built to analyze the modal parameters, and the modal frequencies calculated from the FE model are listed in Table 3.

**Figure 18:**
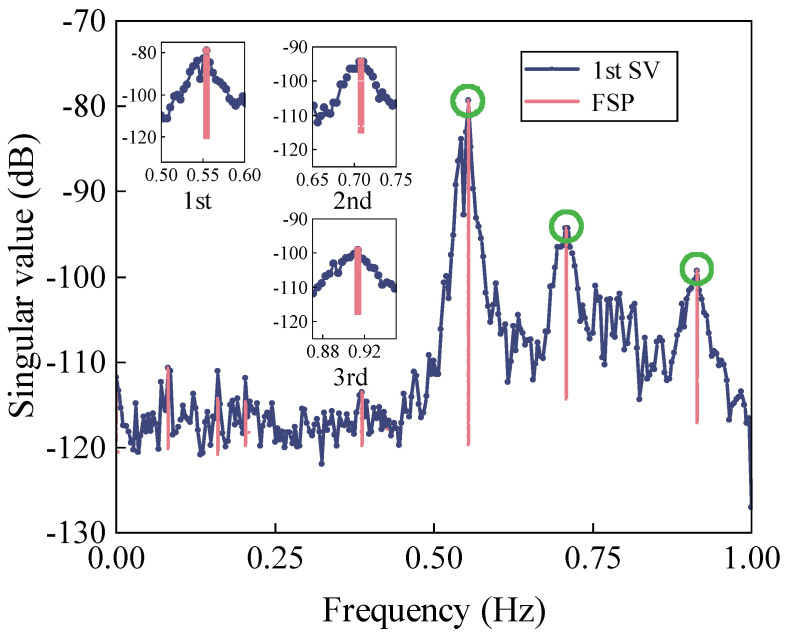
1st SV spectrum after FSP.

As can be seen, this structure has a large number of closely-spaced modes, which makes it difficult to identify the modal parameters. To highlight the modes of interest, the acceleration data were down-sampled from 15.625 Hz to 2 Hz. There were a total of 2304 samples in each measurement. The identified frequencies of the first three dominant modes were 0.55 Hz, 0.71 Hz, 0.91 Hz, respectively, as shown in Figure 18.

**Figure 19:**
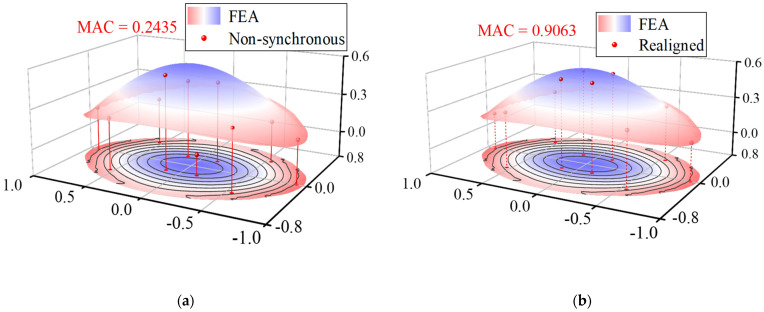

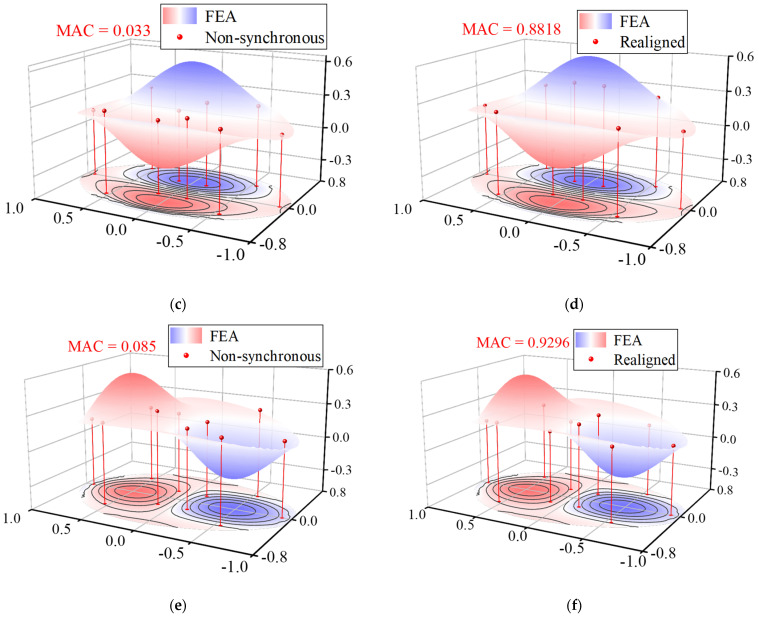
Mode shapes of the first 3 modes of NSSO identified from aligned acceleration and realigned acceleration: (**a**) 1st mode-aligned, (**b**) 1st mode-realigned, (**c**) 2nd mode-aligned, (**d**) 2nd mode-realigned, (**e**) 3rd mode- aligned, and (**f**) 3rd mode-realigned.

The corresponding mode shapes were extracted from the proposed measurement responses. It is found that there is a difference between the theoretical and the identified modal frequencies, which may be caused by the stiffness degradation induced by the cable relaxation. Based on the prior knowledge of the WSNs, the relative lag ranges from −5 s to 5 s. The estimated time delay between the first output and the rest outputs was calculated in sequence as −0.6814 s, 2.8492 s, 3.7431 s, 1.1204 s, 2.2698 s, −0.7102 s, −0.9444 s, −0.2650 s, 0.9082 s by the presented approach. Then the time axis of the WASs was shifted according to the estimated time lags. Ideally, the mode shape components at a symmetric location of sensor placement should have approximately symmetric or anti-symmetric properties. Although a previous synchronization measurement response is best to serve as a reference for comparison, none of the responses are guaranteed to be synchronous due to an inborn deficiency of non-synchronization in such long-range transmission by WSNs. Therefore, the mode shapes calculated from the FE model were set as the reference. For comparison, the modes shapes extracted from the responses before and after the shifted time axis are plotted in Figure 19, along with the mode shapes obtained from the FE model. It can be seen that the mode shape seems to be erratic before the time axis shifts compared to the FE result. The MAC between the reference mode shapes and the mode shapes extracted from non-synchronous data is calculated to quantify the consistency (Figure 19a,c,e). Among the first three dominant modes, the maximum MAC is no more than 0.25, which indicates that the mode shapes identified by non-synchronous data are not correct. On the contrary, the mode shapes obtained from the data after shifting the time axis appear in a symmetric or anti-symmetric manner (Figure 19b,d,f), and the maximum MAC increases to about 0.9.

**Figure 20:**
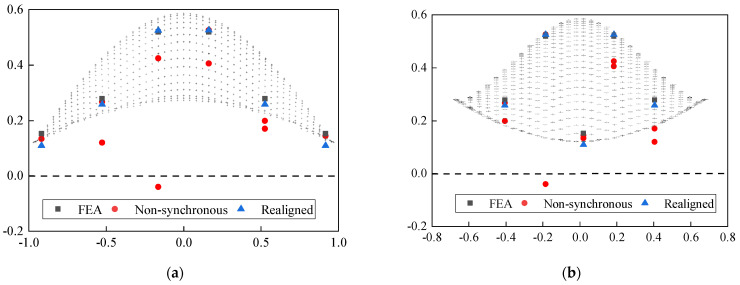

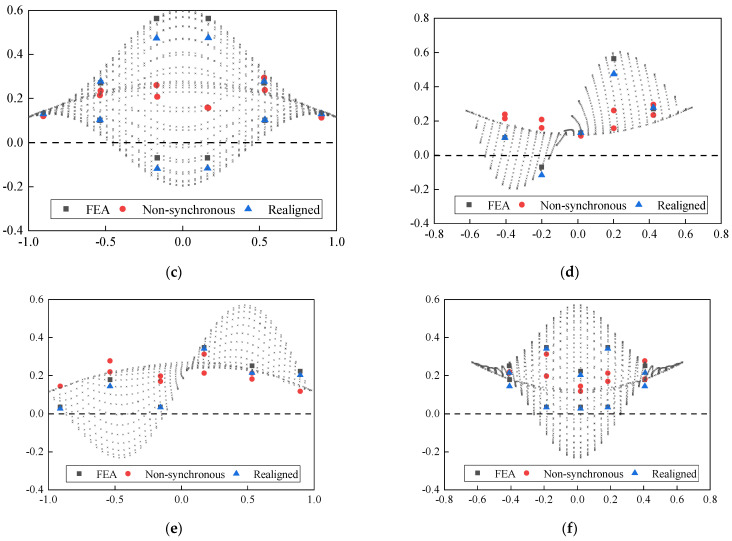
Mode shapes of the first 3 modes of NSSO identified from aligned acceleration and realigned acceleration: (**a**) E-W Projection of 1st mode, (**b**) N-S Projection of 1st mode, (**c**) E-W Projection of 2nd mode, (**d**) N-S Projection of 2nd mode, (**e**) E-W Projection of 3rd mode, and (**f**) N-S Projection of 3rd mode.

To better show the effectiveness of this proposed approach, two orthogonal vertical projections in the north-south and east-west directions of these mode shapes are introduced in Figure 20. The results show that the mode shapes extracted from the processed data are closer to the theoretical results than those obtained from the unprocessed data, which further demonstrates the practicality of the proposed time synchronization approach.

## 5. Conclusions

This study proposes a new time synchronization approach by extending the frequency domain decomposition (FDD) technique. When fed with asynchronous vibration measurements, this data-driven approach that is only based on output fulfills integrated estimation of time lags and identification of modal properties. The relative time lag identified by using lower modes can be regarded as a conservative estimate of the true relative time lag. The Frequency-squeezing processing (FSP) is used in the modal identification by FDD technique to reduce the influence of noise and to improve the readability of the power spectrum representation. A candidate pool of the lags is obtained, and the lags can be further determined by minimizing their standard deviation. Three cases of simulation, experimental test, and field measurement are employed to demonstrate and validate this approach, including the non-synchronous output of a four-story building subjected to white noise excitation, the misaligned acceleration measurements of a five-floor steel frame struck by the Hollister earthquake, and the non-synchronous dynamic record of the National Speed Skating Oval caused by a strong wind. 

The application of this time synchronization approach presupposes that at least two modes need to be identified so that the relative time delay can be uniquely quantified. The accuracy of the time delay estimation is incrementally related to the higher modes obtained through the non-synchronous dynamic measurement responses. The analysis results of the presented three cases show that the proposed time synchronization approach is effective and helps improve the performance of modal identification in WSNs applications.

## Figures and Tables

**Table 1:** Channel delays.

Channel No.	CH 2	CH 3	CH 4
Delay (s)	−0.24	0.68	−0.54

**Table 2:** Comparison between estimated and artificially misaligned time lags.

No.	Time Lag (s)	CH2	CH3	CH4	CH5
1	estimated	0.2692	−0.8023	0.4475	−0.0946
	exact	0.2656	−0.8047	0.4453	−0.0938
	RPE	**1.36%**	**−** **0.3%**	**0.49%**	**0.85%**
2	estimated	0.9175	−0.0289	0.6016	−0.7179
	exact	0.9141	−0.0313	0.6037	−0.7188
	RPE	**0.37%**	**−** **7.67%**	**−** **0.35%**	**−** **0.13%**
3	estimated	−0.0953	0.0023	0.0022	0.0008
	exact	0	0	0	0

**Table 3:** FE model-derived modal frequencies.

Mode No.	Frequency (Hz)	Mode No.	Frequency (Hz)	Mode No.	Frequency (Hz)
1st	0.6097	5th	1.1276	9th	1.3983
2nd	0.8162	6th	1.1924	10th	1.4191
3rd	0.9140	7th	1.1935	11th	1.4357
4th	0.9597	8th	1.3057	12th	1.5471

## Data Availability

Not applicable.
